# Antibiofilm Activity of Chilean Propolis on *Streptococcus mutans* Is Influenced by the Year of Collection

**DOI:** 10.1155/2015/291351

**Published:** 2015-07-12

**Authors:** Jorge Jesús Veloz, Nicolás Saavedra, Alexis Lillo, Marysol Alvear, Leticia Barrientos, Luis A. Salazar

**Affiliations:** ^1^Center of Molecular Biology and Pharmacogenetics, Scientific and Technological Bioresource Nucleus (BIOREN), Universidad de La Frontera, Avenida Francisco Salazar, 01145 Temuco, Chile; ^2^Departamento de Ciencias Químicas y Recursos Naturales, Facultad de Ingeniería y Ciencias, Universidad de La Frontera, Avenida Francisco Salazar, 01145 Temuco, Chile

## Abstract

The chemical composition of propolis varies according to factors that could have an influence on its biological properties. Polyphenols from propolis have demonstrated an inhibitory effect on *Streptococcus mutans* growth. However, it is not known if different years of propolis collection may affect its activity. We aimed to elucidate if the year of collection of propolis influences its activity on *Streptococcus mutans*. Polyphenol-rich extracts were prepared from propolis collected in three different years, characterized by LC-MS and quantified the content of total polyphenols and flavonoids groups. Finally, was evaluated the antibacterial effect on *Streptococcus mutans* and the biofilm formation. Qualitative differences were observed in total polyphenols, flavones, and flavonols and the chemical composition between the extracts, affecting the strength of inhibition of biofilm formation but not the antimicrobial assays. In conclusion, chemical composition of propolis depends on the year of collection and influences the strength of the inhibition of biofilm formation.

## 1. Introduction

The propolis is a resinous substance collected by honeybees (*Apis mellifera*) used to protect the beehive against the invasion of various pathogenic microorganisms. The main bioactive components of propolis are flavonoids, terpenes, and phenolics compounds. However, it is also composed by sugars, hydrocarbons, and mineral elements [1, 2]. Chemical studies have determined a correlation between the composition of propolis with the season and geographic region of collection, plant sources used for collection, and the bee species involved in the process. Thus, its variable composition may have an influence on the biological properties demonstrated by different extracts [3–6]. The pharmacological properties of propolis are well documented and include previous reports of our group describing antidiabetogenic, antiatherogenic, antimicrobial, and antifungal activities of Chilean propolis extracts [7–10] using well characterized extracts in which pinocembrin appears among its main constituents [11, 12].

*Streptococcus mutans* (*S. mutans*) is considered a key player involved in the development of dental caries. Its main virulence is derived from the ability to synthesize water-insoluble and soluble glucans from sucrose, leading to the accumulation of these glucans in a dental biofilm [13], process mediated by the expression of extracellular glucosyltransferases enzymes (GtfS), that in combination with glucan-binding proteins (GBPs) are important for the sucrose-dependent adhesion to the tooth surfaces [14]. The inhibitory capacity of Chilean propolis on the growth of* S. mutans* has been demonstrated but with high variability depending on the characteristics of the extract evaluated [11]. However, the effect of a propolis sample collected in the same geographical place but in different years has not been evaluated. Thus, the aim of the present study was to evaluate the chemical composition and the effect on* Streptococcus mutans* growth and biofilm formation of polyphenol-rich extracts from Chilean propolis collected at the same apiary and same season along three different years.

## 2. Materials and Methods

### 2.1. Preparation of Crude Extracts from Chilean Propolis (CEP)

To evaluate the effect of propolis-collecting year on the chemical composition and antimicrobial activity of Chilean propolis, three propolis samples were obtained from the Andean region of La Araucanía, Chile, in the spring of the years 2008, 2010, and 2011, to prepare three polyphenol-rich extracts (CEP1, CEP2, and CEP3, resp.). Propolis crude samples were kept frozen (−20°C) and protected from light until when propolis polyphenols were simultaneously extracted and analyzed. Frozen propolis samples were crushed to obtain a powder propolis. Then, 30 g was dissolved in 70% ethanol and macerated for 7 days at room temperature. Finally, the solutions were filtered using a whatman paper number 2 and centrifuged at 327 g for 20 minutes to eliminate the resins from the extract.

### 2.2. Determination of Phenolic Compounds Groups

#### 2.2.1. Determination of Total Polyphenols

The content of total polyphenols was quantified using the Folin-Ciocalteu method [15]. CEP (100 *μ*L) was mixed with distilled water (100 *μ*L), Folin-Ciocalteu reagent (2 mL), and sodium carbonate 20% w/v (3 mL). The resultant solution was incubated for 2 hours at room temperature and the absorbance measured in a spectrophotometer (Infinite 200 PRO NanoQuant) at 760 nm. Concentrations were obtained from a calibration curve and expressed as mg mL^−1^ equivalent to the gallic acid.

#### 2.2.2. Determination of Flavones and Flavonols

The content of flavones and flavonols was measured as previously described [16]. CEP samples were diluted 1 : 10 in ethanol 70% (v/v) and 250 *μ*L of this extract was added to 250 *μ*L of aluminum trichloride 5% (v/v) in methanol. The absorbance of the solution was measured at 425 nm in a spectrophotometer (Infinite 200 PRO NanoQuant). Flavonoid concentrations were calculated from a calibration curve and expressed in mg mL^−1^ equivalent to quercetin.

#### 2.2.3. Determination of Flavanones and Dihydroflavonols

Polyphenol-rich extracts were diluted 1 : 10 in ethanol 70% (v/v). Afterward, 0.5 mL of diluted extract was added to 2 mL of 2.4-dinitrophenylhidrazine (DNP), incubated at 50°C for 50 min, and then decanted [17]. Absorbance was measured at 495 nm and the concentration of flavanones and dihydroflavonols was obtained from a calibration curve. Results were expressed in mg mL^−1^ equivalent to pinocembrin used as calibration solution.

### 2.3. Chemical Characterization

To identify the compounds present in the polyphenol-rich extracts we used Liquid Chromatography-tandem Mass Spectrophotometry (LC-MS). For the chromatographic separation RP-C18 Inersil ODS-3 column (2.1 × 150 mm, 3 mm) was used, with 10 *μ*L of injection volume and a flow of 0.2 mL min^−1^ at 35°C. Standards and samples separation were performed using a gradient elution. The eluents A and B were formic acid (0.1%) and methanol, respectively. Flavonoids were studied in negative and positive polarity using the Multiple Reaction Monitoring (MRM) mode and data was acquired through the software Analyst 1.5.1 (Applied Biosystems, USA). In positive polarity, the flavonoids were optimized using standards of apigenin, daidzein, genistein, kaempferol, myricetin, pinocembrin, quercetin, and rutine (Sigma-Aldrich, St. Louis, MO) using the method of direct injection. In the negative polarity, the flavonoids and phenolic acids were optimized using the MRM mode with the p-coumaric acid, ferulic acid, chlorogenic acid, caffeic acid phenethyl ester (CAPE), caffeic acid, and gallic acid as standards (Sigma-Aldrich, St. Louis, MO).

### 2.4. Antimicrobial Activity Testing

Clinical isolates of* Streptococcus mutans* were obtained from the bacterial strain collection of our research center and confirmed by PCR as previously described [18]. Bacteria were grown on Columbia agar plates supplied with sucrose (1%) and incubated in anaerobic atmosphere (Anaerobic Generator GasPak EZ, Becton, Dickinson and Co., NY, USA) at 37°C and for 24 hours. The minimum inhibitory concentration (MIC) and minimum bactericidal concentration (MBC) were determined by the microdilution methodology as described in the Clinical and Laboratory Institute guidelines [19]. Serial dilution tests from the three extract were performed, sterilized in a filters of 0.2 *μ*m, with different total polyphenols concentrations (0.1–100 *μ*g mL^−1^) using an inoculum of 5 × 10^5^ UFC mL^−1^ in sterile trypticase soy broth (TSB) supplied with 1% sucrose, and incubated at 37°C and 5% CO_2_ atmosphere. Sensibility tests were made by triplicate for each extract. Negative controls without treatments and vehicle were also tested.

### 2.5. Biofilm Formation by* Streptococcus mutans* under CEP Treatment

Biofilm growth was quantified by crystal violet staining assay [20]. The* Streptococcus mutans* inoculum (5 × 10^5^ UFC mL^−1^) was incubated at 37°C and 5% CO_2_ atmosphere for 24 hours in 96-well microplates. Attachment cells were grown in microplates with TSB and sucrose (1%) with different total polyphenols concentrations ranging from 0.1 to 100 *μ*g mL^−1^. First, the broth was removed and the plates were washed three times using PBS to eliminate no adherent bacteria and dried at 60°C for 45 minutes. Then, cells were stained using a crystal violet 1% (w/v) solution, incubated for 15 minutes and finally washed with sterile PBS to eliminate the excess of stain. Biofilm formation was determined by adding ethanol 95% to solubilize the crystal violet retained by the cells and optical density (O.D) was measured at 590 nm.

### 2.6. Statistical Analysis

Statistical analysis was performed using the program GraphPad Prism, version 5.0 (US). ANOVA was used for comparison of continuous variables (MBC and MIC). Tukey's Multiple Comparisons posttest was applied when we observed significant differences in ANOVA test, and Dunett's multiple comparisons to compare with the control. The significance level was *α* = 0.05.

## 3. Results

### 3.1. Determination of Different Groups of Phenolic Compounds

Differences were observed in the content of phenolic compounds between the extracts collected along the 3 years. Total polyphenols contained in the CEP2 were superior to CEP1 and CEP3 (*p* < 0.0001) and the content of flavones and flavonols differed between the three extracts analyzed. No differences were observed regarding the concentration of flavanones and dihydroflavonols (*p* = 0.228). Quantifications of phenolic compounds in polyphenol-rich extracts from Chilean propolis are listed in Table 1.

### 3.2. Chemical Characterization

The chemical characterization of three CEP obtained by LC/MS using both retention times and spectra transitions in positive and negative polarity distinguished flavonoids and phenolic acids. The majority of compounds are present in the three analyzed extracts; however, there are some qualitative variations. Among the common flavonoids compounds are apigenin, genistein, kaempferol, myricetin, pinocembrin, and quercetin. Daidzein and rutine were detected depending on the year of collection (Table 2). Regarding phenolic acids, CAPE, caffeic, p-coumaric, and ferulic acids were detected in all extracts analyzed. Chlorogenic and gallic acids were dependent on the year (Table 3).

### 3.3. Antimicrobial Testing

Antibacterial activity of the analyzed extracts was tested determining MIC and MBC in* Streptococcus mutans* cultures under treatment with polyphenols. Both parameters showed no variations between the extracts collected in different years (Table 4; MIC, *p* = 0.177; MBC, *p* = 0.645).

### 3.4. Biofilm Formation by* Streptococcus mutans*

**Figure 1:**
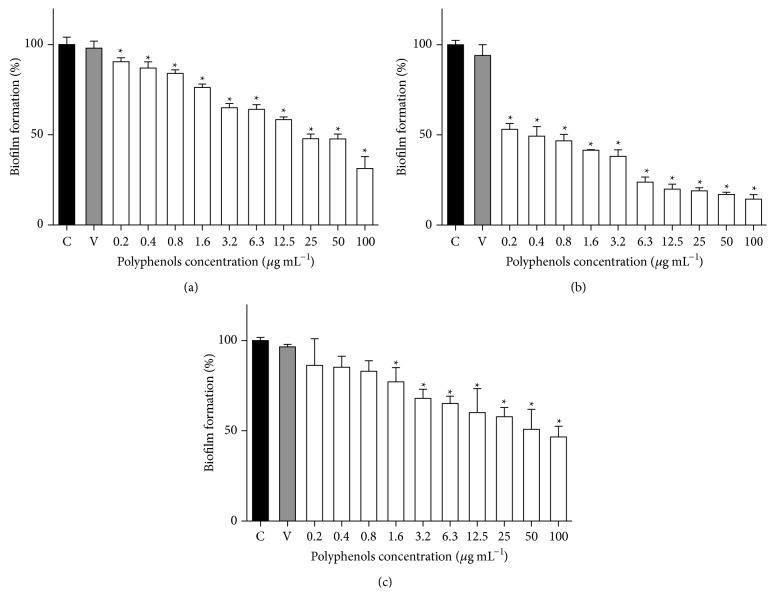
Biofilm formation in* Streptococcus mutans* cultures treated with polyphenol-rich extracts from Chilean propolis. (a), (b), and (c) figures show the effect on biofilm formation of CEP1, CEP2, and CEP3, respectively. C: control; V: vehicle. ANOVA: *p* < 0.0001,  ^*∗*^Dunett's multiple comparisons versus control: *p* < 0.05.

The effect of CEP on biofilm formation by* Streptococcus mutans* was determined by the Crystal Violet staining assay. The growth of bacterial plaque diminished with dose-dependent effects, starting from 0.2 *μ*g mL^−1^ for the CEP1 and CEP2. The CEP3 showed a less potent effect with an inhibitory effect starting from 1.6 *μ*g mL^−1^ (Figure 1).

## 4. Discussion

The medicinal properties of propolis have been widely described and include* Streptococcus mutans* antibacterial capabilities, suggesting the use of propolis as a cariostatic agent [21]. Biological activities of polyphenol-rich extracts from propolis show variations depending on its chemical composition, which in turn depends on its geographical origin, botanical sources, and the season of collection [22]. Thus, propolis samples from the same regions might have similar types of flavonoids and other phenolic compounds [23]. We prepared polyphenolic-rich extracts from Chilean propolis samples collected during spring at the same apiary along three different years. These samples were obtained from a nontranshumant apiary, so it is expected that vegetal species contributing to the production of propolis in the hive should not vary significantly between each year of production. Previously reported data characterizing the botanical origin of Chilean propolis from La Araucanía showed that* Lotus uliginosus* Schk. was the predominant vegetal source followed by* Caldcluvia* or* Eucryphia*, whose distinctive elements are not able to differentiate because of their high similarity [11]. Likewise, the plant debris identified in CEP1, CEP2, and CEP3 samples showed a predominance of structures from* Lotus uliginosus *Schk. (58–61%) and* Caldcluvia* or* Eucryphia* (19–23%).

Colorimetric assays were performed to quantify total polyphenols and flavonoids using two assays to determine flavone and flavonols or total flavanones and dihydroflavonols content. The three extracts analyzed in this study showed differences in total content of polyphenols depending on the year of collection, with the highest content in the propolis from the spring of 2010 (CEP2). The analyzed extracts had a slightly higher content than previously reported for Chilean propolis that have exhibited a high variability according to its geographical origin (from 3.4 to 21.4 mg mL^−1^), with the highest content observed in propolis from La Araucania [11]. The content of flavonoids, flavones, and flavonols also showed differences according to the year of collection and concordantly with the content of total polyphenols, a higher amount was observed in CEP2. However, the qualitative composition of flavonoids obtained by LC-MS showed the CEP2 as the extract with fewer compounds among the studied flavonoids. These findings suggest a quantitative compensation of absent flavonoids by the other compounds identified in the extract, so the quantification of individual compounds is an interesting issue to consider in further analysis.

The composition of the analyzed extracts was similar to that previously described for propolis from La Araucanía in which pinocembrin is the predominant compound [11]. The composition of Chilean propolis includes several classes of flavonoids and phenolic acids. Fragmentation patterns and retention times showed the presence of specifics members of different chemical families as flavones, represented by apigenin; flavonols such as quercetin, kaempferol; flavanones represented mainly by pinocembrin; and isoflavones as daidzein and genistein, whose characteristics ions in positive polarity were corresponding with previous LC-MS spectrum for propolis from other geographical areas [24, 25]. In comparison with propolis from other geographical origin, the Chilean propolis has a chemical composition with a higher diversity of compounds. Poplar types' propolis from Europe, Asia, and North America is characterized by the presence of pinocembrin, pinobanksin, galangin and benzyl, phenethyl, and prenyl caffeates; Northern Russia samples have significant levels of acacetin, apigenin, ermanin, and kaempferide. Another propolis collected from tropical areas, such as the Brazilian green propolis, contains prenylated p-coumaric acids, prenylated acetophenones, and diterpenic acids [6, 22, 26]. Cinnamic acid derivatives were the most abundant phenolics acids, including caffeic, p-coumaric, and ferulic acid and were present in the samples, but gallic acid was detected only in the CEP1, similarly to tropical and subtropical sources rich in p-coumaric acids and diterpenic and triterpenic acids [15]. Moreover, chemical composition of propolis has been linked to its botanical origin, which corresponds to the botanical sources from which bees produce propolis. Data previously reported for Chilean propolis have shown variations depending on the month of collection [27]. Similar results were described for the content of total polyphenols and flavonoids for propolis from Argentina [28]. The samples analyzed in the present study were collected during the same month along three different years, which could mean that the predominant botanical resources are maintained between samples and thus its chemical composition is only slightly affected. In brief, our results show some quantitative and qualitative variations in the composition of the polyphenol-rich extracts from Chilean propolis collected on the years 2008, 2010, and 2011, which could influence its biological activities.

When we analyzed the antibacterial activity using both MIC and MBC assays, no differences were observed between the three polyphenol-rich extracts. This finding suggests that antibacterial activity of the polyphenol-rich extracts analyzed results from the action of common compounds present in these three extracts as apigenin; a flavonoid identified in all CEP samples showed antimicrobial activity in previous studies [29], although the mechanisms of antimicrobials properties of propolis have not been completely elucidated. As pointed out previously, it can also be associated with a synergistic effect of its components [21]. Finally, the inhibitory effect of polyphenol-rich extracts on biofilm formation was variable between the three extracts. Concordantly with the observed in the content of polyphenols and flavonoids, the second extract (CEP2) showed a highest inhibition at lower concentrations of polyphenols, starting from 0.2 *μ*g mL^−1^ with an inhibition of about 50% on biofilm formation. Other authors obtained similar results in microplates assays starting with 100 *μ*g mL^−1^ of EEP and observed decreased biofilm formation percentages and dose-dependent effects [30]. Glucosyltransferases B and C (GtfB and GtfC) play a key role on biofilm formation in oral cavity. Several reports show multiple inhibitory activities at concentrations as low as 25 *μ*g mL^−1^ of 6 propolis types and an effective inhibition of GtfB and GtfC, affecting the process of dental caries and plaque formation [31, 32]. Therefore, this may be a possible responsible mechanism of the effect of polyphenol-rich extracts on biofilm formation by* Streptococcus mutans*.

## 5. Conclusion

In summary, our results indicate that polyphenol-rich extracts from Chilean propolis present qualitative differences in its composition that are dependent on the year of collection. The year-related content differences distinctively inhibit the biofilm formation of* Streptococcus mutans*. However, they do not have an influence on bacterial growth.

## Figures and Tables

**Table 1:** Influence of propolis-collecting year on the content of phenolic compounds groups in polyphenol-rich extracts.

Group of compounds	CEP1	CEP2	CEP3	^*∗*^ *p*-value
Total polyphenols, mg mL^−1^	24.6 ± 0.4^a^	29.0 ± 0.8^b^	24.7 ± 0.2^a^	**<0.0001**
Flavones and flavonols, mg mL^−1^	10.2 ± 0.03^a^	11.9 ± 0.05^b^	9.8 ± 0.1^c^	**<0.0001**
Flavanones and dihydroflavonols, mg mL^−1^	8.3 ± 0.3	9.4 ± 0.6	8.2 ± 1.3	0.228

CEP1, CEP2, and CEP3: polyphenol-rich extracts from Chilean propolis collected in 2008, 2010, and 2011, respectively. Results expressed as mean ± standard deviation. Total polyphenols, flavones, and flavonols and flavanones and dihydroflavonols are expressed as gallic acid, quercetin, and pinocembrin equivalent, respectively. ^*∗*^ *p* value from ANOVA test. Different letters indicate significant differences after Tukey's Multiple Comparisons posttest.

**Table 2:** Flavonoids identified in three polyphenol-rich extracts from Chilean propolis by LC-MS.

Compound	CEP1	CEP2	CEP3	Retention time	MW	Main fragments
Apigenin	+	+	+	42.6	270	269, 254, 226, 167
Daidzein	+	N.D.	+	40.5	254	153, 129
Genistein	+	+	+	44.4	270	253, 215
Kaempferol	+	+	+	34.6	286	269, 241, 229, 183
Myricetin	+	+	+	30.0	318	301, 273, 169, 153
Pinocembrin	+	+	+	42.0	256	239, 215, 173, 153
Quercetin	+	+	+	32.5	302	285, 257
Rutine	+	N.D.	N.D.	35.9	309	300, 271

CEP1, CEP2, and CEP3: polyphenol-rich extracts from Chilean propolis collected in 2008, 2010, and 2011, respectively. + indicates presence; N.D.: not detected.

**Table 3:** Phenolic acids identified in three polyphenol-rich extracts from Chilean propolis by LC-MS.

Compound	CEP1	CEP2	CEP3	Retention time	MW	Main fragments
Caffeic acid	+	+	+	15.3	180	135, 105
CAPE	+	+	+	41.8	284	139, 135
Chlorogenic acid	N.D.	+	+	30.9	354	191, 161
P-coumaric acid	+	+	+	16.7	164	119, 104
Ferulic acid	+	+	+	34.5	194	178, 134
Gallic acid	+	N.D	N.D	8.5	170	125, 107

CEP1, CEP2, and CEP3: polyphenol-rich extracts from Chilean propolis collected in 2008, 2010, and 2011, respectively. CAPE: caffeic acid phenethyl ester; + indicates presence; N.D.: not detected.

**Table 4:** Antimicrobial activity of polyphenol-rich extracts from Chilean propolis on *Streptococcus mutans*.

Antimicrobial activity	CEP1	CEP2	CEP3	^*∗*^ *p* value
MIC *μ*g mL^−1^	0.91 ± 0.59	0.22 ± 0.15	0.39 ± 0.35	0.177
MBC *μ*g mL^−1^	1.30 ± 0.44	1.05 ± 0.44	0.91 ± 0.59	0.645

CEP1, CEP2, and CEP3: polyphenol-rich extracts from Chilean propolis collected in 2008, 2010, and 2011, respectively. MIC: minimum inhibitory concentration; MBC: minimum bactericide concentration. Results expressed as mean ± standard deviation. ^*∗*^ *p* value from ANOVA test.
